# Exploring potential decreasing age of patients with frontal fibrosing alopecia

**DOI:** 10.1016/j.jdin.2022.07.002

**Published:** 2022-07-22

**Authors:** Monica Rosales Santillan, Jared B. Goldberg, Lynne J. Goldberg

**Affiliations:** a Department of Dermatology, Boston University School of Medicine, Boston Medical Center, Boston, Massachusetts; b Consumer Edge, New York, New York

**Keywords:** age, alopecia, association, cobalt, cosmetic, FFA, frontal fibrosing alopecia, genetic, hair loss, sunscreen

*To the Editor:* Frontal fibrosing alopecia (FFA) is a primary scarring alopecia that primarily affects postmenopausal women. Since the first report of FFA in the 1990s, its incidence and prevalence have increased.^1^^,^^2^ While the etiology of this disease is still unknown, patient demographics may help identify risk factors for development.

The reported mean age of FFA onset is 56-63 years.^1^ In 2019, the senior author evaluated 2 patients with FFA in their 20s and hypothesized that patient age of onset is decreasing. With IRB approval, a retrospective review of patients seen in the Hair Clinic at Boston Medical Center was undertaken to further investigate the age at which patients were presenting with FFA.

Patients diagnosed with FFA from 2005 to 2019 were tabulated, and charts from 2012 to 2019 (when electronic medical records became available) were reviewed. Data collected were patient sex, age at time of initial visit, and, when available (2012 on), estimated age at disease onset. Ordinary least squares regression was used to analyze trends (Table I) using R version 3.6.3 (R Core Team, 2020). Two-tailed *P* < .05 indicated significance. Of 292 patients, 282 (96.6%) were female. Age at time of visit ranged from 24 to 84 years, and estimated age of disease onset ranged from 17 to 81 years. Both decreased over time, but neither achieved statistical significance. The age of the youngest patient presenting annually significantly decreased over time (*P* < .001, Fig 1). A regression analysis did not reveal that patients sought care earlier over time.

**Table I:** Descriptive statistics and regression results for frontal fibrosing alopecia patients over time annually

Patient characteristic	*N*	Range	Mean	SD	β	*P*
Age (in years) at time of initial visit (2005-2019)	292	24-84	59.3	11.2	−0.127	.550
Age of youngest patient at time of initial visit (2005-2019)	15	24-53	37.3	9.3	−1.643	<.001
Age at time of estimated disease onset (2012-2019)	250	17-81	55.4	11.3	−0.236	.465
Delay in seeking care (age at initial visit minus age at time of estimated disease onset, 2012-2019)	250	0-16	3.7	2.9	0.135	.103

*n* = Number of patients; *SD* = standard deviation; β = regression coefficient.

**Fig 1:**
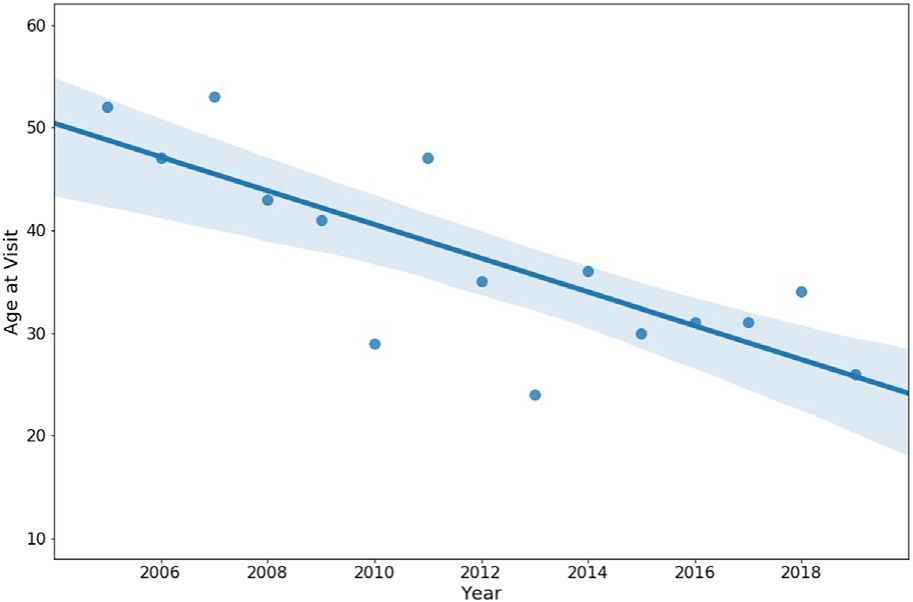
Youngest patients with frontal fibrosing alopecia on initial presentation over time (β = −1.643, *P* < .001).

The cause of FFA is unknown. Hypotheses include decreased estrogen levels, allergic contact dermatitis, environmental factors, genetic factors, and cosmetic surgical procedures. Our findings of younger patients with FFA over time, as well as a French report of 3 children with FFA,^3^ suggests that estrogen levels and cosmetic surgical procedures may not be the only determinants. Cobalt chloride hexahydrate, a metal found in various products including facial skin care products, was the most frequent allergen found in a multicenter study on FFA.^4^ Multiple observational studies have evaluated the role of sunscreen as an environmental factor; however, there are insufficient data currently to support this association.^5^ Facial moisturizers and occupational exposures are also suspect. It is plausible that younger patients are presenting due to earlier exposure to more environmental insults, though our findings may also be due to an increased awareness of the diagnosis and shifts in treatment-seeking behaviors in the general population over time.

Our patients averaged 55.4 years at disease onset, which is younger than that in a recent analysis.^1^ Strikingly, there was a statistically significant decrease in the youngest patient age seen annually over time. Perhaps because older patients continue to be affected, onset in younger patients is being masked by the wide age range of affected patients. An additional study limitation was the sample size from 2005 to 2007, when few patients with FFA were seen. Another was potentially inaccurate patient recollection of disease onset, especially if eyebrows were affected first. Larger cohort studies are needed to further assess patient characteristics that may lead to identifying risk factors for this enigmatic disease.

## Conflicts of interest

None disclosed.

## References

[1] Porrino-Bustamante M.L., Fernandez-Pugnaire M.A., Arias-Santiago S. (2021). Frontal fibrosing alopecia: a review. J Clin Med.

[2] Mirmirani P., Tosti A., Goldberg L., Whiting D., Sotoodian B. (2019). Frontal fibrosing alopecia: an emerging epidemic. Skin Appendage Disord.

[3] Atarguine H., Hocar O., Hamdaoui A. (2016). Frontal fibrosing alopecia: report on three pediatric cases. Arch Pediatr.

[4] Rudnicka L., Rokni G.R., Lotti T. (2020). Allergic contact dermatitis in patients with frontal fibrosing alopecia: an international multi-center study. Dermatol Ther.

[5] Robinson G., McMichael A., Wang S.Q., Lim H.W. (2020). Sunscreen and frontal fibrosing alopecia: a review. J Am Acad Dermatol.

